# Itraconazole Reverts ABCB1-Mediated Docetaxel Resistance in Prostate Cancer

**DOI:** 10.3389/fphar.2022.869461

**Published:** 2022-06-03

**Authors:** Thiago S. Lima, Luciano O. Souza, Diego Iglesias-Gato, Johanna Elversang, Flemming Steen Jørgensen, Tuula Kallunki, Martin A. Røder, Klaus Brasso, José M.A. Moreira

**Affiliations:** ^1^ Department of Drug Design and Pharmacology, Faculty of Health and Medical Sciences, University of Copenhagen, Copenhagen, Denmark; ^2^ CAPES Foundation, Ministry of Education of Brazil, Brasília, Brazil; ^3^ Sino-Danish Center for Education and Research, Aarhus University, Aarhus, Denmark; ^4^ Department of Pathology, Rigshospitalet, Copenhagen University Hospital, Copenhagen, Denmark; ^5^ Cancer Invasion and Resistance, Danish Cancer Society Research Center, Copenhagen, Denmark; ^6^ Department of Urology, Copenhagen Prostate Cancer Center, Center for Cancer and Organ Disease—Rigshospitalet, Copenhagen University Hospital, Copenhagen, Denmark; ^7^ Department of Clinical Medicine, University of Copenhagen, Copenhagen, Denmark

**Keywords:** metastatic castration-resistant prostate cancer, docetaxel resistance, cellular models, androgen independence, drug repurposing

## Abstract

Docetaxel (DTX) was the first chemotherapeutic agent to demonstrate significant efficacy in the treatment of men with metastatic castration-resistant prostate cancer. However, response to DTX is generally short-lived, and relapse eventually occurs due to emergence of drug-resistance. We previously established two DTX-resistant prostate cancer cell lines, LNCaP^R^ and C4-2B^R^, derived from the androgen‐dependent LNCaP cell line, and from the LNCaP lineage-derived androgen-independent C4-2B sub-line, respectively. Using an unbiased drug screen, we identify itraconazole (ITZ), an oral antifungal drug, as a compound that can efficiently re-sensitize drug-resistant LNCaP^R^ and C4-2B^R^ prostate cancer cells to DTX treatment. ITZ can re-sensitize multiple DTX-resistant cell models, not only in prostate cancer derived cells, such as PC-3 and DU145, but also in docetaxel-resistant breast cancer cells. This effect is dependent on expression of ATP-binding cassette (ABC) transporter protein ABCB1, also known as P-glycoprotein (P-gp). Molecular modeling of ITZ bound to ABCB1, indicates that ITZ binds tightly to the inward-facing form of ABCB1 thereby inhibiting the transport of DTX. Our results suggest that ITZ may provide a feasible approach to re-sensitization of DTX resistant cells, which would add to the life-prolonging effects of DTX in men with metastatic castration-resistant prostate cancer.

## Introduction

Despite continued medical advances, prostate cancer (PCa) still claims the lives of over 375,000 men globally each year (Sung et al., 2021). Androgen deprivation therapy (ADT) is the mainstay of systemic therapy for locally advanced and metastatic PCa. Although ADT is efficacious, many patients will eventually experience disease progression and develop castration-resistant prostate cancer (CRPC). Patients with metastatic CRPC (mCRPC) have poor prognosis and very limited treatment options; clinical management of mCRPC involves essentially cytotoxic chemotherapy and androgen-targeted therapies. Docetaxel (DTX), a microtubule-stabilizing taxane, was the first chemotherapeutic agent to have shown significant clinical benefit in the treatment of mCRPC in two randomized phase III studies, SWOG 99-16 and TAX327 (Petrylak et al., 2004; Tannock et al., 2004). The survival benefit demonstrated in these trials was modest (2–3 months) but significant. Recently, novel therapeutic options including third-generation hormone therapy agents, or combinatorial regimens have improved the clinical management of advanced PCa. Hormonal agents, such as abiraterone acetate, an inhibitor of cytochrome P45017A1 (CYP17A1), targeting androgen biosynthesis (Barrie et al., 1994), or enzalutamide, which targets the androgen receptor (AR) (Tran et al., 2009), have been shown in randomized trials to improve overall survival when given either before (Scher et al., 2010; Rathkopf et al., 2014) or after DTX (Fizazi et al., 2012).

DTX remains a standard of care for progressing patients with mCRPC, even though about 50% of patients do not respond initially to the therapy and those that do, will eventually fail treatment due to development of resistance (Canil and Tannock, 2004). Drug resistance in cancer is a well-known phenomenon and the main limiting cause to drug efficacy. In fact, multidrug drug resistance (MDR) constitutes a major clinical problem in cancer treatment (Holohan et al., 2013). Previous studies have identified multiple mechanisms of resistance, through which PCa becomes resistant to docetaxel. Two of the most common mechanisms are: enhanced intracellular drug extrusion activity mediated by members of the family of adenosine triphosphate-binding cassette (ABC)-transporters, such as ABCB1/MDR1, and aberrant AR signaling (van Brussel and Mickisch, 2003; Holohan et al., 2013). ABCB1 is an ATP-dependent efflux pump, which decreases the intracellular concentration of a variety of anti-cancer drugs, such as doxorubicin, vincristine, actinomycin-D, paclitaxel and DTX, leading to MDR in several types of cancer, including prostate cancer (Szakács et al., 2006; Sánchez et al., 2009). Inhibition of ABCB1 efflux activity has been shown to reverse the MDR phenotype in model systems (Amin, 2013; Kadioglu et al., 2016), but clinical development of ABCB1 inhibitors has been largely unsuccessful, primarily due to lack of efficacy and/or development of severe side effects (Shukla et al., 2011). Although four generations of ABCB1 inhibitors have now been developed, their clinical value has been limited by prohibitive non-specific toxicity issues (Robey et al., 2018).

We have recently established two DTX-resistant PCa cell models, based on LNCaP and C4-2B sub-lines (LNCaP^R^ and C4-2B^R^, respectively), and characterized them in terms of mechanisms of resistance to DTX (Lima et al., 2021). These lines constitute a cellular model of PCa progression that mimics the natural history of the disease, and we used them to look for drugs that could prevent resistance to DTX. Results of genome-wide mRNA microarray analysis of these two resistant cell lines identified, among others, upregulation of the *ABCB1* gene, which encodes a transmembrane glycoprotein, P-glycoprotein (P-gp), known to be involved in drug resistance. We have previously implemented a strategy in drug development based on biomarker-guided repurposing of chemotherapeutic drugs for cancer therapy (Stenvang et al., 2013). Drug repurposing provides a cost- and time-effective approach to drug development based on the use of existing drugs in new indications (Turanli et al., 2018; Xu and Bubley, 2019). We used our DTX-resistant cell lines to screen known drugs to find one that could revert or bypass drug-resistance in LNCaP^R^ and C4-2B^R^, respectively. We found that itraconazole (ITZ), a triazole anti-fungal drug widely used in the prevention and systemic treatment of a broad range of fungal infections, could reverse ABCB1-mediated DTX resistance. This effect was observed in other resistant cells, not only in prostate cancer derived cells, such as PC-3 and DU145, but also in DTX-resistant breast cancer cells. Overexpression of efflux pumps leads to resistance to a series of anticancer drugs in various cancers and constitutes a major challenge in cancer therapy. Our results suggest that ITZ may exert life-prolonging reversal of resistance by blocking ABC transporters in multidrug resistant cancer cells.

## Methods

### Cell Culturing and Reagents

Sub-lines with acquired drug resistance to docetaxel (C4-2B^R^ and LNCaP^R^, respectively) were generated as previously described (Lima et al., 2021). C4-2B^R^ and LNCaP^R^ showed docetaxel IC_50_ values of 99.47–100.50 nmol/L and 49.50–50.65 nmol/L, respectively, compared to the C4-2B and LNCaP parental cells that had IC_50_ values of 1.00–1.40 nmol/L, and 0.78–1.06 nmol/L, respectively (Lima et al., 2021). DU145 parental and docetaxel-resistant DU145^R^, and PC-3 parental and docetaxel-resistant PC-3^R^ prostate cancer cells (Ellegaard et al., 2013), were kindly provided by Marja Jaättellä (University of Copenhagen, Denmark). All PCa cells were cultured and maintained in RPMI-1640 medium + glutaMAX™-I (Gibco, Invitrogen, Carlsbad, CA, United States) supplemented with 10% fetal bovine serum (FBS). MDA-MB-231 parental and DTX-resistant MDA-MB-231^R^, and MCF-7 parental and DTX-resistant MCF-7^R^ breast cancer cells (Hansen et al., 2016), were kindly provided by Jan Stenvang. MDA-MB-231 and MCF-7 cells were cultured and maintained in Dulbecco’s modified Eagle’s medium (DMEM) including L-glutamine, supplemented with 10% and 1% fetal bovine serum (FBS), respectively. The clear cell renal cell carcinoma line RCC-FG2, and breast ductal carcinoma lines HCC1419, HCC1569, HCC202, and BT483 (ATCC, United States) were cultured and maintained in RPMI-1640 medium supplemented with 2 mM L-glutamine and 10% fetal bovine serum. All cell lines were tested for mycoplasma contamination using Mycoplasma PCR detection kit (GATC-Biotech, Köln, Germany). The FDA-approved drug library (SCREEN-WELL FDA-approved drug library V2) was from Enzo Life Sciences (Farmingdale, NY, United States). Itraconazole was acquired from Sigma-Aldrich (Sporanox; Merck Life Science A/S, Denmark).

### Cytotoxicity Assay

Cytotoxicity was performed using the 3-(4,5-dimethylthiazol-2-yl)-2,5-diphenyl-tetrazolium bromide (MTT) assay as previously described (Carmichael et al., 1987). Briefly, cells were plated at a density of 8,000 cells/well in triplicates in 96-well cell culture plate and allowed to grow in drug free medium over 24 h prior to treatment. After 48 or 72 h of treatment, drugs were removed and 0.5 mg/ml MTT (Sigma-Aldrich) was added to each well. Following incubation for three hours, 20% sodium dodecyl sulphate (SDS) in 0.02 M hydrochloric acid (HCL) was added to each well to dissolve the formed formazan crystals overnight. Absorbance of formazan was measured in a microplate spectrophotometer (PowerWaveX, Bio-Tek Instruments, INC.) at 570 nm and the background absorbance of MTT was measured at 670 nm. Cell viability was expressed in percent relative to untreated control cells.

### Protein Extraction and Western Blotting

Whole-cells were harvested and lysed using lysate buffer M-PER Mammalian Protein Extraction Reagent (Thermo Scientific) supplemented with Pierce Protease and Phosphatase Inhibitor Mini tables (Thermo Scientific). Cell lysates were centrifuged at 14,000 g for 10min at 4°C and supernatants collected. Total amount of protein was assessed by the Pierce™ BCA Protein Assay Kit (Thermo Scientific), according to manufacturer’s instructions. The Novex^®^ NuPAGE^®^ MES SDS Running Buffer (Thermo Fisher Scientific) was used for separation of proteins according to manufacturer’s instructions. Samples were loaded onto precast 10- or 15- well 4–12% Bis-Tris Gel gels (Novex^®^ NuPAGE^®^, Invitrogen). Proteins were blotted onto a nitrocellulose membrane (iBlot^®^2 NC, Invitrogen) using iBlot^®^2 gel transfer device. Blots were blocked for 1 h in washing buffer (PBS+0.1% Tween 20) containing 5% non-fat dry milk and incubated overnight with appropriate primary antibody diluted in blocking reagent: Anti-MDR1 (Abcam, Cambridge, United Kingdom); Purified Mouse Anti- β-actin (BD Transduction Laboratories™, NJ, United States). After washed for 3 × 10 min in TBS-T, membranes were incubated with horseradish peroxidase-conjugated secondary antibody (Mouse/Rabbit) for 1 h at RT. Membranes were further washed 3 × 10 min in TBS-T and developed using Clarity Western ECL substrate (Bio-Rad) detection reagent. Proteins bands were detected with UVP BioSpectrum Imaging System (UVP, CA, United States).

### Tissue Microarray

The set of TMA blocks analysed by immunohistochemistry comprised samples from a consecutive series of men (*n* = 336), with clinically localized prostate cancer who underwent radical prostatectomy with curative intent from 1 January 2002 until 31 December 2005 at the Department of Urology, Rigshospitalet, Copenhagen, Denmark. Detailed construction of the TMA blocks and clinicopathological parameters of the samples has been previously reported (Kristensen et al., 2018). Briefly, two 1-mm cores from each of a representative malignant and non-malignant tissue areas were mounted on a total of 44 TMA blocks. The study was approved by the Danish National Committee on Health Research Ethics for the Capital Region (J.nr. H-6-2014-111).

### Immunostaining

Freshly cut 2.5 µm sections of each of 44 TMA blocks were deparaffinized, rehydrated and exposed to thermal-induced antigen retrieval to unmask epitopes. Sections were boiled for 10 min in Envision Flex Target Retrieval Solution, high pH (Dako, Glostrup, Denmark) diluted 1:50 in miliQ H_2_O, before being incubated for 1 h with primary MDR1 antibody (Abcam #ab170904) diluted 1:1,000 in antibody diluent with background reducing components (Dako). Then sections were washed twice in TBS + 0.5% Triton X-100 and incubated for 20 min with High Definition Polymer Detector (AH diagnostics). Colorimetric signals were detected using DAB. Sections were developed with EnvisionTM FLEX DAB + Chromogen (Dako) diluted in EnvisionTM FLEX Substrate Buffer to visualize the primary antibody. Sections were counterstained with Mayer’s hematoxylin (Hounisen, Skanderborg, Denmark) and mounted with Pertex xylene-based mounting media (Hounisen). Positive controls for MDR1 staining consisted of tissue cores from normal liver and FFPE-embedded docetaxel resistant cells (LNCaP^R^ and C4-2B^R^, respectively).

### Rhodamine 123 Efflux Assay

The rhodamine 123 (Rho123; Sigma-Aldrich) efflux assay was used to assess functional activity of ABCB1 in the presence or absence of ITZ. Briefly, cells (5 × 10^5^) were incubated at 37°C in the dark for 30 min in DMEM medium supplemented with 5% FCS and containing 0.5 μmol/L Rho123. Cells were then washed extensively with ice-cold PBS to completely remove extracellular Rho123, and subsequently incubated in the presence or absence of ITZ 2.5 μmol/L in DMEM at 37°C for 90 min. As a control, Valspodar (PSC-833; 1 μmol/L), a selective P-glycoprotein inhibitor, was used instead of ITZ. Cells were then washed with ice-cold PBS and immediately analyzed for intracellular Rho123 fluorescence on a FACSort flow cytometer. Mean fluorescence intensity (MFI) was calculated for each analysis. Five thousand events were analyzed.

### Docetaxel Intake Imaging and Quantification

To examine the cellular behavior of DTX in the presence of ITZ, we performed *in vivo* imaging of drug resistant C4-2B^R^ and LNCaP^R^ cells and the corresponding parental lines C4-2B and LNCaP. For imaging, 9–12,000 cells were seeded into black, 190 μm clear bottom 96-well microplates (Screenstar, Greiner). Two days later cells were treated, as indicated, with ITZ or vehicle together with 0.5 μM nuclear violet (AAT Bioquest) and 0.1 μM DTX^DeepRed^ (Tubulin Tracker DeepRed, Invitrogen), which consists of DTX conjugated with a bright and stable far-red fluorescent dye. One hour later cells were set up for live imaging using ImageXpress Micro confocal high-throughput microscope (Molecular Devices) and scanned and imaged every two hours, until the final 7-h timepoint using widefield mode and a ×40 objective, in 5% CO_2_ atmosphere and 37°C. Nuclear violet was imaged with a DAPI filter (Ex. 377/54nm, Em. 447/60) and DTX^DeepRed^ with a Cy5 filter (Ex. 631/28nm, Em. 692/40 nm). The images were analyzed using MetaXpress software (Molecular Devices) and the cell scoring program using the violet nucleus as an indicator of a cell to calculate the % of cells with far-red dye incorporated to their tubulin network. Results presented are representative of three independent experiments.

### Molecular Docking

The recently determined three-dimensional structures of ABCB1 from *Cyanidioschyzon merolae* (CmABCB1, PDB entries 6A6M and 6A6N) were retrieved from the Protein Data Bank and used as model for the human ABCB1 (Kodan et al., 2019). After water molecules and ligands were deleted, the Protein Preparation Wizard in Maestro version 11.1 was used to prepare the protein structures for docking by adding missing side-chains, assigning proper atom types and bond orders, and optimizing the hydrogen-bonding network pH = 7.0 (Sastry et al., 2013). A restrained minimization of the proteins was performed with convergence of heavy atoms to RMSD = 0.30 Å using the OPLS3e force field (Roos et al., 2019). The structure of ITZ, the (2R,4S,2′R)-stereoisomer, was retrieved from PubChem (entry CID 55283). ITZ is a weak base with pKa values of the triazole and piperazine moieties predicted by the MarvinSketch version 15.4.20 program from ChemAxon (https://www.chemaxon.com) to be 2.2 and 3.9, respectively. Possible tautomers and protonation states were generated at pH 7.0 ± 2.0. The different stereoisomers were generated in Maestro and subjected to a short energy minimization. The GOLD (Genetic Optimisation for Ligand Docking) program version 5.6 was used for docking of ITZ to both the homodimeric structures and the monomeric structure (A-chain) of ABCB1 (Jones et al., 1997). Binding sites were defined by a 30 Å sphere centered at a residue in the binding cavity (Glu530 in 6A6M and Tyr 358 in 6A6N). Ligands were docked with the slow genetic algorithm and using ChemScore as scoring function (Verdonk et al., 2003). Ten poses were sampled and analyzed for each docking. The Prime program implemented as part of the Schrodinger software system version 2019-4 (Schrödinger, LLC, New York, NY, 2014, http://www.schrodinger.com) was used for determination of the MM/GBSA free energies of binding (Li et al., 2011; Genheden and Ryde, 2015). The program yields free energies of binding based on the actually provided as well as on relaxed target and ligand structures (Table 2, ΔG Bind and ΔG Bind(NS), respectively).

### Statistical Analysis

Comparison of dose-response curves was performed by non-linear regression fitting of curves with a built-in model [log (inhibitor) vs. response] using GraphPad Prism software (GraphPad Prism 9, GraphPad Software, United States). For each dataset we then used ANOVA to test for significance in differences in best-fit values in IC_50_ of the dataset, with IC50 values are the same for all data sets as null hypothesis. Significance level was set to 5%.

## Results

### Expression of ABCB1 in Chemo-Naïve PCa Patients

**FIGURE 1:**
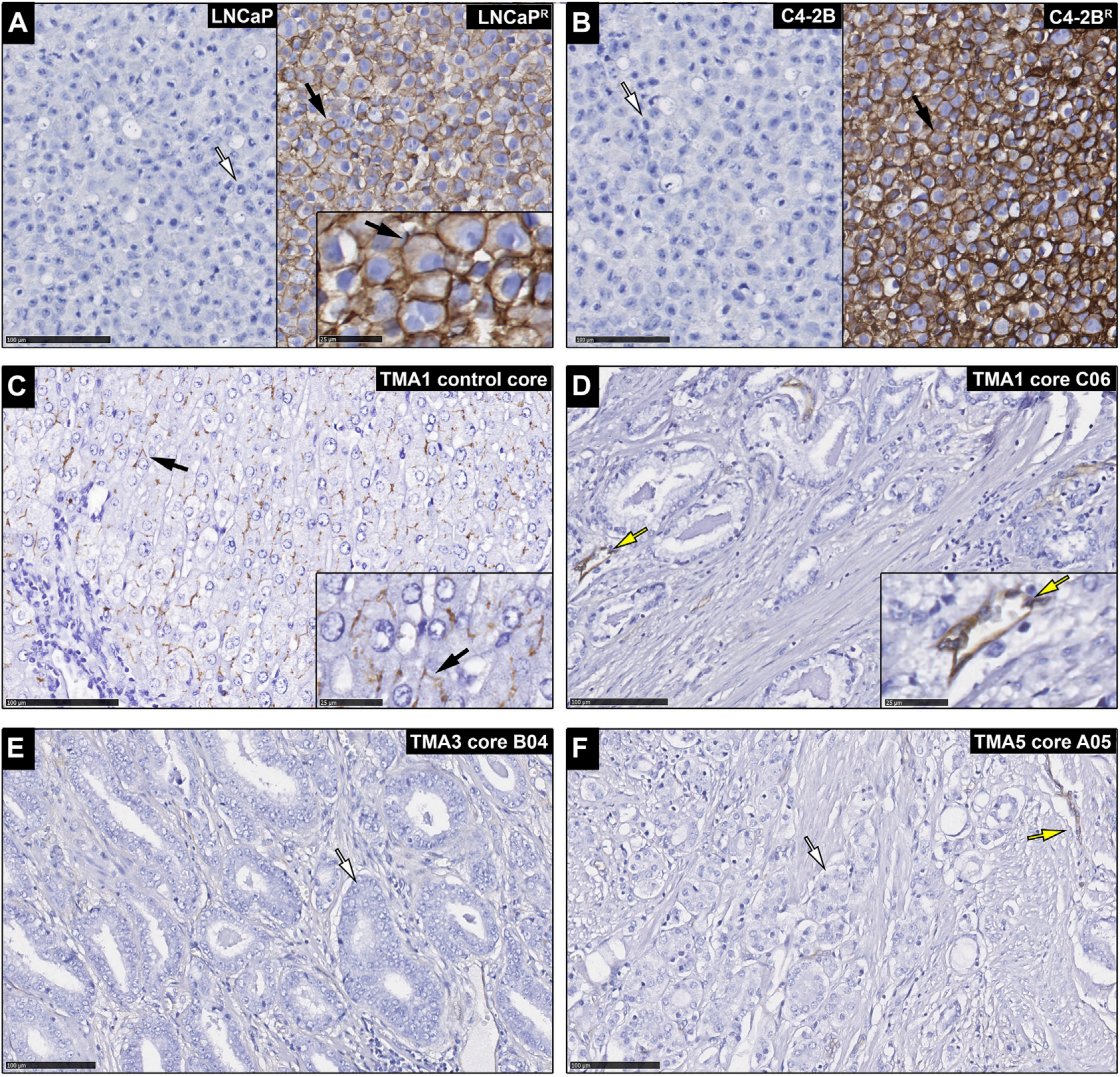
Expression of ABCB1 in chemo-naïve prostate cancer patients. Immunohistochemical analysis of ABCB1 expression in **(A)** LNCaP cells (left panel) with white arrow indicating a cell with no immunostaining, and LNCaP^R^ cells (right panel) with black arrow indicating a cell with ABCB1 immunostaining (inset is a higher magnification image with black arrow showing cell membrane staining). **(B)** C4-2B cells (left panel) with white arrow indicating a cell with no immunostaining, and C4-2B^R^ cells (right panel) with black arrow indicating a cell with ABCB1 immunostaining. **(C)** Normal liver tissue with black arrow indicating membrane immunostaning (control core; inset is a higher magnification image with black arrow showing cell membrane staining). **(D–F)** prostate carcinomas with glandular cells showing no immunoreactivity (white arrows) and staining observed only in blood vessels (yellow arrows). Scale bars, 100 and 25 µm in inset images.

Although up-regulation of *ABCB1* expression is expected to occur following exposure of cancer cells to DTX, thus underpinning acquisition of resistance to chemotherapy, high levels of expression of *ABCB1* may also contribute to intrinsic resistance to DTX in chemo-naïve tumors. Then, if that was the case one could use expression of ABCB1 as a predictive biomarker. Because cancer samples are an admixture of many different cell types, and there were conflicting reports of *ABCB1* predictive value depending on whether studies were done at the mRNA or protein level (Vergier et al., 1993), we performed an immunohistochemistry (IHC) study of ABCB1 expression in a large cohort of chemo-naïve prostate cancer tissue samples. In this way we could assess the specific expression of ABCB1 protein (P-gp) in cancer cells, as well as the relative expression of the membrane-bound functional drug transporter. Prostatectomies from 336 chemo-naïve castration resistant prostate cancer (CRPC) patients (Kristensen et al., 2018) (Table 1), were used to assess the potential of ABCB1 expression as a predictive biomarker for DTX resistance in prostate cancer (Figure 1). We found that apart from endothelial cells (yellow arrows; Figures 1D,F), PCa samples were negative for ABCB1 expression (white arrows; illustrated in Figures 1E,F). As expected, the positive controls, FFPE embedded LNCaP^R^ and C4-2B^R^ cells, and normal liver tissue (black arrows; Figures 1A–C, respectively), showed ABCB1 staining. FFPE embedded LNCaP and C4-2B cells showed no immunoreactivity for ABCB1 (Figures 1A,B, left panel, respectively). Regardless of the functional importance of ABCB1 in MDR in PCa, our data suggested that ABCB1 may not be useful as a predictive biomarker of intrinsic DTX resistance in chemo-naïve CRPC patients.

**TABLE 1:** Clinicopathologic parameters of patients included in the TMA.

	Study population *n* = 315
Age at baseline, years, median (IQR)	62.8 (59.3–66.5)
Neoadjuvant treatment	
No	308 (97.8%)
Yes	7 (2.2%)
PSA, µg/L, median (IQR)	10.0 (6.8–15.0)
Clinical T-Stage	
cT1	159 (50.5%)
cT2a/b/c	149 (47.3%)
cT3a/b	7 (2.2%)
Biopsy Gleason Score	
≤6	215 (76.5%)
3 + 4	47 (16.7%)
4 + 3	5 (1.8%)
08–10	14 (5.0%)
Missing	34
Radical Prostatectomy	
≤6	124 (39.4%)
3 + 4	112 (35.6%)
4 + 3	51 (16.2%)
08–10	28 (8.9%)
Pathological T-stage	
pT2a/b/c	203 (64.4%)
pT3a/b	112 (35.6%)
N-stage	
N0/x	309 (98.1%)
N1	6 (1.9%)
Margin Status	
R-	131 (41.6%)
R+	184 (58.4%)
ERG	
Negative	120 (38.1%)
Positive	195 (61.9%)

Abbreviations, IQR: inter quartile range; PSA, prostate specific antigen.

### Itraconazole Can Overcome Drug-Resistance in DTX-Resistant PCa Cells

**FIGURE 2:**
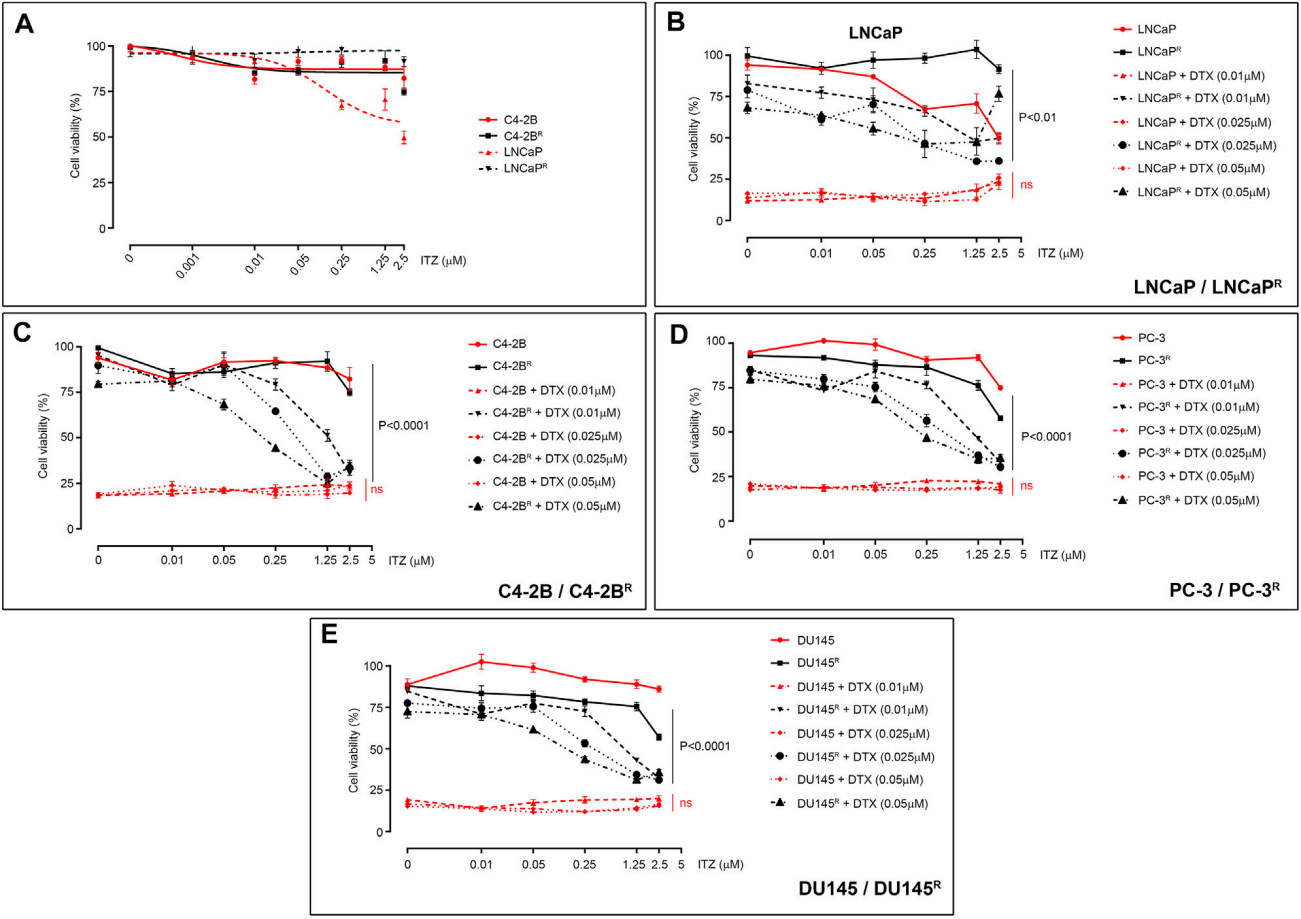
Effect of ITZ on PCa cells. **(A)** Effect of ITZ on LNCaP, C4-2B, LNCaP^R^ and C4-2B^R^ cells, with only LNCaP showing dose-dependent cytotoxicity. Combining increasing concentrations of ITZ with DTX showed dose-dependent reversal of drug-resistance in DTX-resistant PCa cell lines **(B)** LNCaP^R^, **(C)** C4-2B^R^, **(D)** PC-3^R^ and **(E)** DU145^R^. Cell viability was measured using a standard MTT assay, following exposure of cells to varying concentrations of docetaxel for 72 h. All plotted values are normalized to the untreated control. Significance in differences of best-fit IC_50_ values for the different ITZ + DTX combinations were tested by two-way ANOVA. ns: non-significant *p*-value.

Currently, drug development strategies targeting ABCB1-mediated drug resistance in cancer mostly combine ABCB1 inhibitors with substrate drugs to re-establish drug sensitivity in the drug-resistant cells. We used our DTX-resistant cell models to screen a library of 786 FDA-approved drugs (Screen-Well FDA-approved drug library V2) for compounds (drug X, 10 µM) that could kill the DTX-resistant C4-2B^R^ cells in combination with 0.01 μM DTX. We found that one drug, itraconazole (ITZ), showed little cytotoxicity of its own, with only a modest effect observed at higher concentrations (>2.5 μM; Figure 2A), however, ITZ was able to efficiently kill DTX-resistant cells when combined with DTX. Given that ITZ has a favorable toxicity profile and can be used for long-term maintenance treatment (Persat et al., 1992), should the combination of ITZ with low doses of DTX efficiently overcame drug resistance it may be of clinical value. To assess the effect of ITZ on DTX resistance, LNCaP^R^ and C4-2B^R^ cells were treated with various concentrations of DTX combined with ITZ for 72 h, and cell viability was assessed by MTT (Figures 2B,C). Addition of ITZ showed a dose-dependent reversal of sensitivity to DTX in LNCaP^R^ (Figure 2B, *p* < 0.01) and C4-2B^R^ cells (Figure 2C, *p* < 0.0001), respectively.

Unlike other azole antifungal drugs, such as fluconazole and ketoconazole, which are known to block gonadal and adrenal steroidogenesis (Patel et al., 2018; Munkboel et al., 2019), ITZ is not expected to interfere with androgen synthesis. To determine if the effect of ITZ on drug resistance was independent of androgen receptor (AR) activity, we investigated if ITZ could reverse DTX resistance in AR-negative ABCB1-overexpressing prostate cancer cell lines. We treated two previously described AR-negative DTX-resistant cell lines, PC-3^R^ and DU145^R^, with DTX in combination with ITZ for 72 h (Figures 2D,E, respectively). Cell viability was assessed by MTT and, as shown in Figure 2, AR signaling was not a determining factor as ITZ could also revert DTX resistance in these cells in a dose-dependent manner (Figures 2D,E; *p* < 0.0001 and *p* < 0.0001, respectively).

### Itraconazole Reversal of Drug-Resistance Is Associated With ABCB1 Expression and Cancer-independent

**FIGURE 3:**
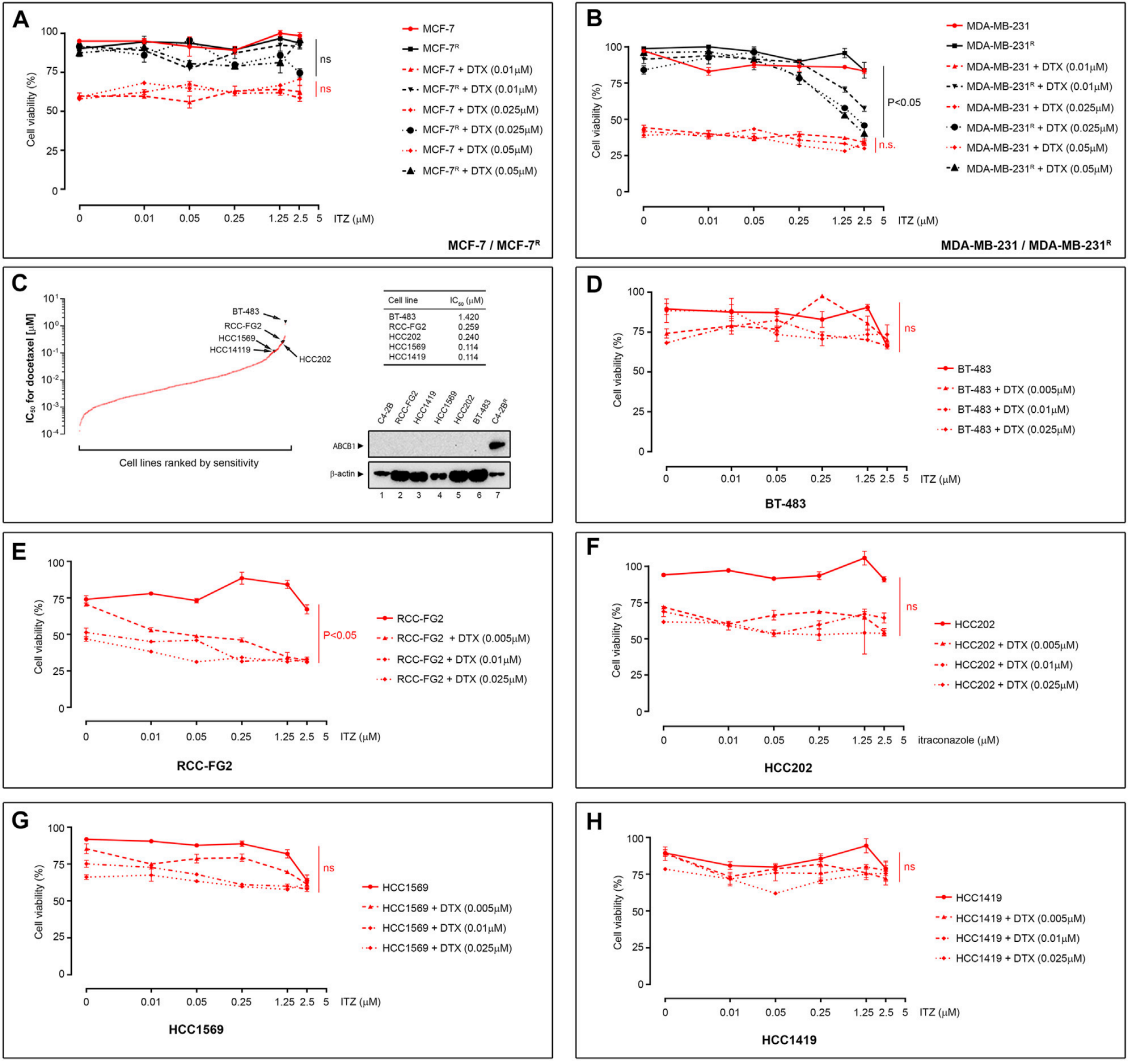
Effect of ITZ on DTX-resistant cancer cells. **(A)** Effect of ITZ on DTX-resistant MCF-7^R^ and **(B)** MDA-MB-231^R^ cells breast cancer cell lines. Combining increasing concentrations of ITZ with DTX showed dose-dependent reversal of drug-resistance in DTX-resistant PCa cell lines **(C)** Plotted IC_50_ values for DTX Genomic of Drug Sensitivity in Cancer project platform dataset. Five cells lines showing no ABCB1 expression (western blot analysis inset) were selected and the effect of ITZ determined alone and in combination with DTX for the **(D)** BT-483, **(E)** RCC-FG2, **(F)** HCC202, **(G)** HCC1569 and **(H)** HCC1419. Cell viability was measured using a standard MTT assay, following exposure of cells to varying concentrations of docetaxel for 72 h. All plotted values are normalized to the untreated control. Significance in differences in best-fit values in the IC_50_ values of the dataset were tested by two-way ANOVA. ns: non-significant *p*-value.

ITZ has been shown to revert daunorubicin resistance in murine leukemia P388/ADR multidrug resistant cells, as well as adriamycin resistance in K562 cells (K562/ADR) and HL60 human leukemia cells (HL60/ADR) (Gupta et al., 1991), suggesting that ITZ may reverse multidrug resistance in various settings. DTX is used not only for treatment of metastatic PCa, but also in other cancers such as breast cancer. We used the DTX-resistant breast cancer cell lines MDA-MB-231^R^ and MCF-7^R^, previously established in our group (Hansen et al., 2016), to evaluate the activity potential of ITZ in breast cancer cells. The MDA-MB-231^R^ and MCF-7^R^ sublines have been previously characterized, and ABCB1 upregulation was identified as a major alteration associated with docetaxel resistance in these cells (Hansen et al., 2016). As shown in Figure 3, ITZ was able to reverse DTX resistance in MDA-MB-231^R^ cells (Figure 3B, *p* < 0.05) but failed to do so on MCF-7^R^ cells (Figure 3A), respectively.

To determine whether ITZ was able to overcome DTX resistance unrelated to ABCB1 upregulation, we analyzed the DTX sensitivity data from the Genomic of Drug Sensitivity in Cancer project platform (GDSC1 dataset) to identify cell lines with substantial intrinsic DTX resistance (IC_50_ > 0.1 µM; Figure 3C). We found five lines that had no detectable expression of ABCB1 (Figure 3C, lower right subpanel). The five cell lines are shown ranked by their sensitivity and respective IC_50_ (Figure 3C, table inset). As previously, all five cell lines were treated with DTX alone (5–25 nM), ITZ alone and a combination of both, for 72 h. As shown in Figure 3D through Figure 3H, BT-483 (Figure 3D), HCC202 (Figure 3F), HCC1569 (Figure 3G), and HCC1419 (Figure 3H) cell lines did not demonstrate significant differences in cell viability when exposed to DTX or ITZ, alone or in combination, respectively at the concentrations tested. The RCC-FG2 cell line showed a borderline significant effect of ITZ in combination with DTX (*p* = 0.0475) (Figure 3E). Taken together, our data suggested that ITZ might only surpass DTX resistance associated with ABCB1 upregulation.

### Itraconazole Blocks ABCB1 Efflux Activity

**FIGURE 4:**
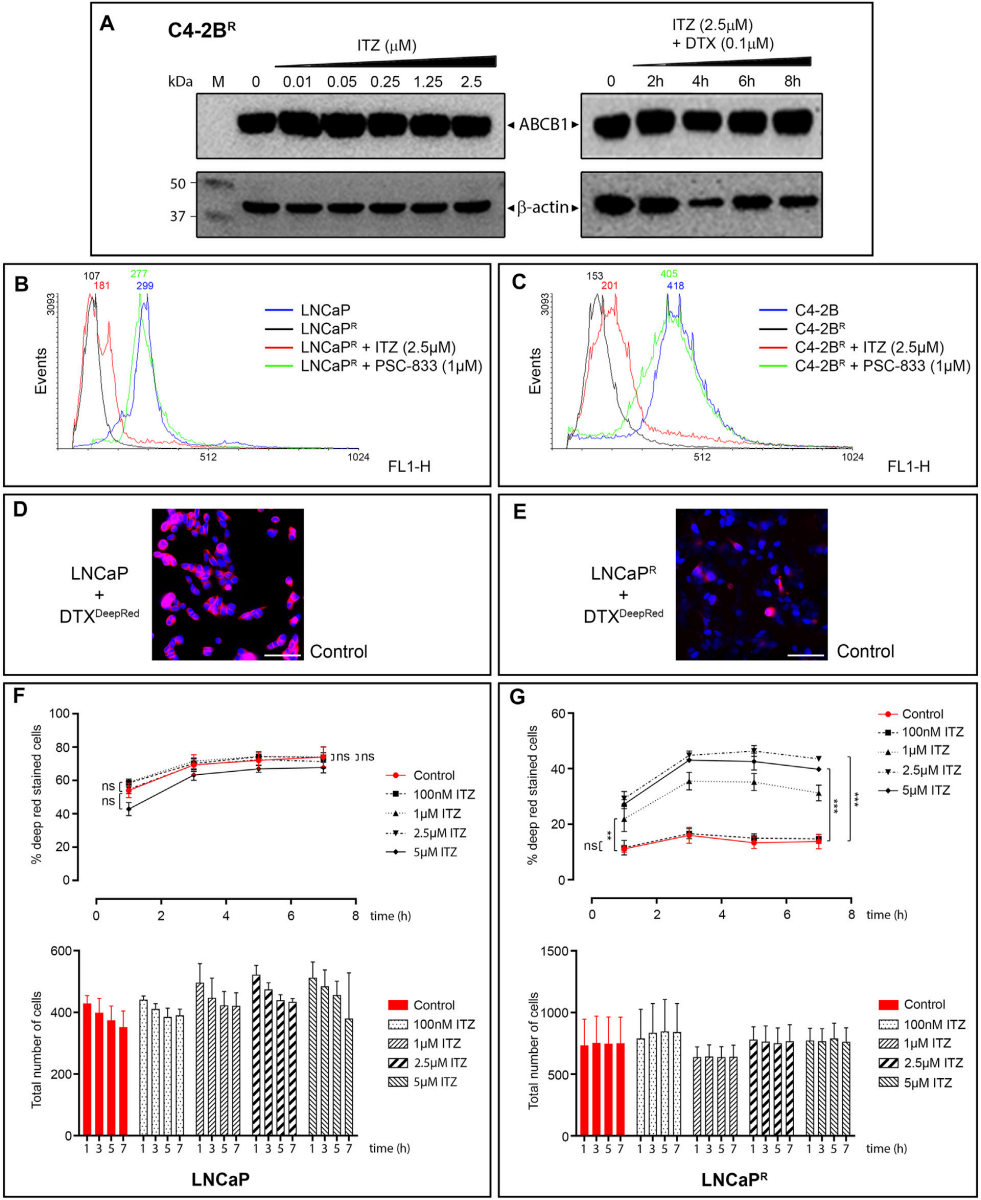
Expression of ABCB1 and *in vitro* modulation of ABCB1-dependent efflux of DTX. **(A)** ABCB1 expression levels in C4-2B^R^ cells. ABCB1 expression on total protein cellular extracts was assessed by Western blot analysis using β-actin expression as normalization factor. ABCB1 expression was evaluated after a 48 h exposure to increasing concentrations of ITZ (left panel) and after a combined treatment with ITZ (2.5 µM) and DTX (100 nM) for up to 8 h (right panel). We found no significant effect of ITZ on ABCB1 expression under these conditions. The effect of ITZ on Rho123 efflux in **(B)** LNCaP^R^ and **(C)** C4-2B^R^ cells was analysed by FACS analysis. Fluorescence histograms of Rho123 accumulation are shown after exposure to ITZ (red line), or to the ABCB1 inhibitor PSC-833 (green line). Levels of Rho123 are also shown for untreated cells (black line) and the non-resistant cell line (blue line). Single-cell *in vivo* imaging of drug resistant **(D)** LNCaP and **(E)** LNCaP^R^ cells exposed to fluorescent DTX^DeepRed^ showed that, in the absence of ITZ (control samples; vehicle treated), DTX^DeepRed^ accumulates in the LNCaP parental cell line but is efficiently extruded from the DTX resistant LNCaP^R^ line. **(F,G)** Cells exposed to various concentrations of ITZ, showed ITZ was able to block efflux of DTX^DeepRed^ in a dose-dependent manner. Bar graphs shows mean ± s.d. of mean fluorescent intensity values for DTX^DeepRed^ efflux from DTX-resistant LNCaP^R^ cells in absence (red bars) or absence of ITZ (black bars), respectively. Scale bars, 80 µm. Significance in differences in best-fit values in the IC_50_ values of the dataset were tested by ANOVA. ns: non-significant *p*-value. ∗∗*p* < 0.01. ∗∗∗*p* < 0.001.

Although ITZ has been reported to be an ABCB1 inhibitor at clinically relevant doses (Wang et al., 2002), the exact mechanism(s) by which it can overcome multidrug resistance remains unclear. ITZ is reportedly a potent inhibitor of the hedgehog (Hh) signaling pathway (Kim et al., 2010; Inoue et al., 2016), and the Hh pathway effector transcriptional factor GLI1 regulates expression of ABCB1 (Chen et al., 2014). To determine whether ITZ modulated expression of ABCB1 in the DTX-resistant PCa cells, we examined the expression of ABCB1 in ITZ treated cells alone or in combination with DTX. We found no effect of ITZ on ABCB1 expression in any of the cell lines tested (illustrated for C4-2B^R^ in Figure 4A). We then evaluated whether ITZ ability to reverse docetaxel resistance stems from inhibition of ABCB1 efflux activity. We analysed the efflux activity of ABCB1 using the cationic fluorescent dye Rhodamine 123 (Rho123) as a tracer. Rho123 is a substrate of ABCB1 and its cellular accumulation provides an useful proxy for evaluating ABCB1-mediated drug-efflux activity (Ludescher et al., 1992). We found that ITZ had no significant effect on Rho123 efflux from LNCaP^R^ and C4-2B^R^ cells (Figures 4B,C). By contrast, the ABCB1 inhibitor valspodar (PSC-833) was able to efficiently block efflux of Rho123 from LNCaP^R^ and C4-2B^R^ cells (Figures 4B,C, green line), respectively. Since it was possible that ITZ affected only some very specific ABCB1 substrates, we examined the levels of intracellular DTX in LNCaP^R^ and C4-2B^R^ cells, in the absence or presence of various concentrations of ITZ (Figures 4C–G). We performed single-cell *in vivo* imaging of drug resistant C4-2B^R^ and LNCaP^R^ cells using DTX conjugated with a bright and stable far-red fluorescent dye (DTX^DeepRed^) in the absence or presence of various concentrations of ITZ. As can be seen in Figure 4, in the absence of ITZ DTX^DeepRed^ accumulates in the LNCaP parental cell line (Figure 4D) but is efficiently extruded from the DTX resistant LNCaP^R^ line (Figure 4E). ITZ was able to block efflux of DTX^DeepRed^ in a dose-dependent manner in LNCaP^R^ cells (Figure 4G), restoring intracellular concentrations of DTX^DeepRed^ to levels comparable, albeit lower, to those of LNCaP cells (Figure 4F). The same effect was observed in C4-2B^R^ cells (data not shown), indicating that ITZ is an ABCB1 inhibitor.

### Molecular Docking Suggests ITZ Can Inhibit ABCB1 Directly

**FIGURE 5:**
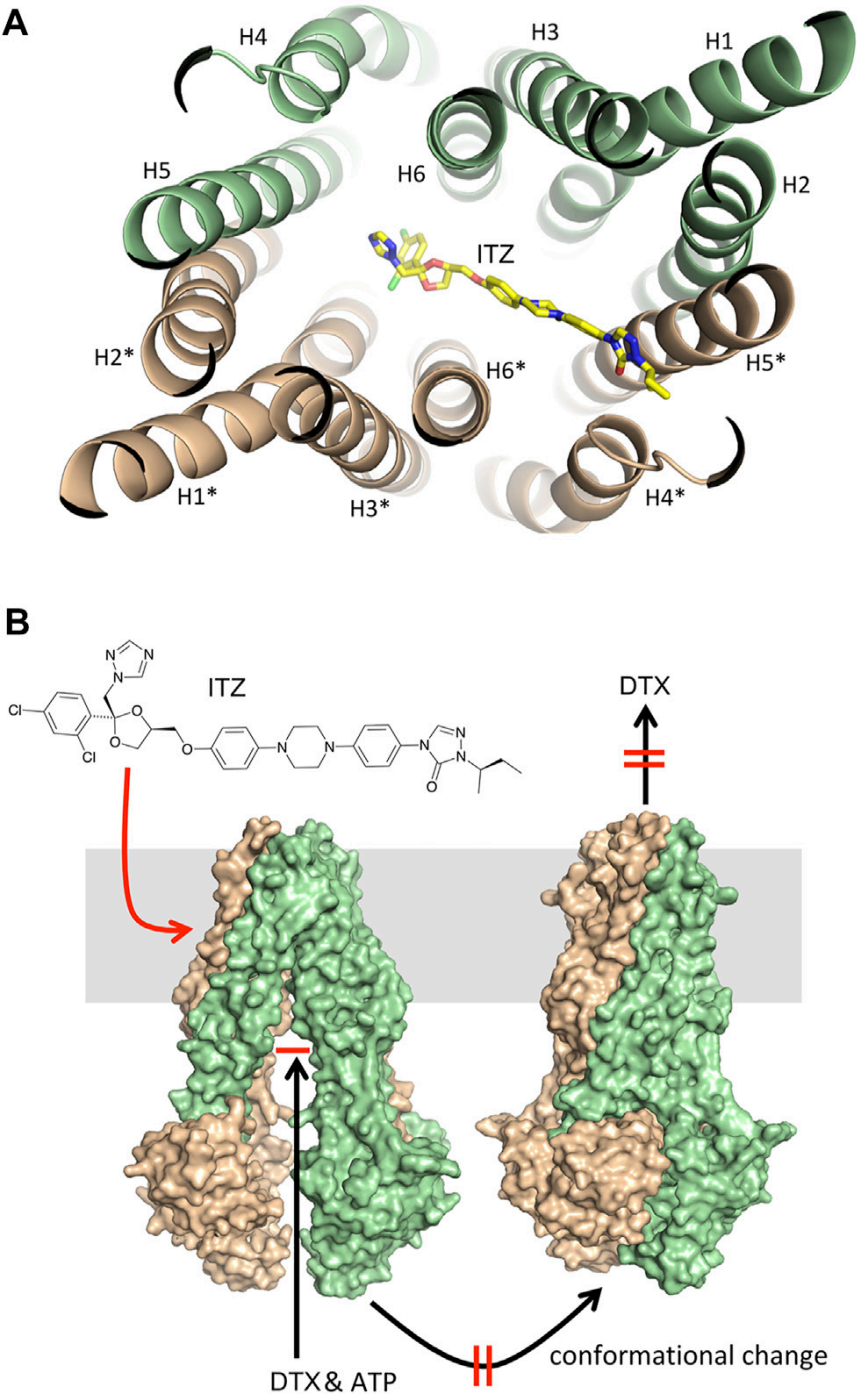
Molecular docking. **(A)** Illustration of the preferred binding mode of itraconazole between the trans-membrane helices in the inward-facing form of P-gp CmABCB1. The helices are shown as light green and beige cartoons numbered analogously with the numbering reported by Kodan and colleagues (Kodan et al., 2019). Itraconazole is displayed as a color-typed stick model. **(B)** Illustration of the P-gp mediated transport of docetaxel involving docetaxel binding to the inward-facing form, the ATP induced conformational change to the outward-facing form and subsequent release of docetaxel (black arrows) and the effect of itraconazole by binding to the inward-facing form, and thereby inhibiting the conformational change and thus the transport of docetaxel across the membrane (red arrow and markings).

Docking of ITZ to the inward-facing form of ABCB1 yielded a series of very similar poses, all indicating that ITZ binds at the trans-membrane part of the transporter between the two monomers (Figure 5A). The dioxolane end of ITZ is sandwiched between helices H6 and H6* with the dichloro-substituted benzene and triazole rings pointing towards H5 and H1*. Side-chains from these helices (H5: Met351, Ile354 and Tyr358; H6: Phe383, Phe384, Ile387 and Met391; H1*: Leu135, Phe138 and Phe142; H6*: Phe384, Ile387, Leu388 and Met391) form a hydrophobic cavity encircling this part of the ITZ structure. Five and eight of the 11 residues interacting with ITZ are identical and similar, respectively, to the corresponding residues in the human ABCB1. The other end of the ITZ molecule with the 1,2,4-triazole-3-one moiety is poking in between H4* and H5*. The only polar interaction we observe is a hydrogen bond between Ser350 on H5* and the carbonyl group in the 1,2,4-triazole-3-one moiety. For comparison, we have also docked ITZ to the inward-facing form of the ABCB1 homodimer and to the ABCB1 monomer to compare the energetics of the different binding scenario. For both proteins ITZ binds close to the ATP-binding sites (data not shown). GOLD binding energies (GOLD ΔG, Table 2) show that binding to the homodimers is more favorable than binding to the monomer, and that binding to the outward-facing form is superior to binding to the outward-facing form. The ranking is further supported by more rigorously determination of the free energy of binding determined by the Schrödinger implementation of the MM/GBSA method (MM/GBSA ΔG, Table 2). ITZ contains three chiral centers and, accordingly, may exist as eight stereoisomers (Shi et al., 2010). The commercial ITZ formulation is a mixture of the four cis-stereoisomers, which recently have been shown not only to be more potent than the trans-stereoisomers, but also to display different hepatotoxicity and antiangiogenic activity (Shi et al., 2010; Shim et al., 2016). Our original studies were done on the (*2R,4S,2′R*)-isomer retrieved from PubChem, but subsequently we have docked all eight stereoisomers to the inward-facing form of the transporter. For all eight stereoisomers we find docking poses displaying binding modes like the one reported for the (*2R,4S,2′R*)-isomer. Generally, we find that the cis-isomers have better GOLD scores than the trans-isomers by an average on 4 kcal/mol. Thus, our docking studies indicate that ITZ binds tightly deep in between the trans-membrane spanning alpha-helices of the inward-facing form of ABCB1 and thereby can inhibit the transport of substrates such as DTX as illustrated in Figure 5B.

**TABLE 2:** Energies for itraconazole binding to P-gp (all values in kcal/mol).

	GOLD score	GOLD ΔG	MM/GBSA ΔG bind	MM/GBSA ΔG bind(NS)
6A6M monomer	29.2	−34.2	−32.7	−46.0
6A6M homodimer, outward-facing	29.8	−37.8	−43.2	−56.4
6A6N homodimer, inward-facing	39.0	−41.3	−56.4	−64.9

## Discussion

Development of resistance is one of the major factors limiting efficacy of chemotherapeutic drugs. Treatment failure can be due to one, or multiple, factors but upregulation of ABC efflux transporters has been proposed as a major common mechanism underlying MDR. We previously generated a set of DTX resistant PCa cell lines (LNCaP^R^ and C4-2B^R^, respectively) showing up-regulation of the ABCB1 drug efflux as a mechanism contributing to the resistance phenotype (Lima et al., 2021). Drug efflux pumps can decrease chemotherapeutic drug efficacy by reducing the intracellular concentration of anticancer drug substrates to sub-therapeutic levels. Although there is strong mechanistic evidence for ABC transporters mediating drug resistance, their possible role in clinical multidrug resistance is still cause for some debate, not least due to the lack of success of ABCB1 inhibitors in clinical trials (Robey et al., 2018). However, several new lines of evidence suggested that ABC transporters may in fact have a role in clinical drug resistance under specific conditions (Robey et al., 2018). We used our DTX-resistant cell sublines to pursue an Rx/CDx drug development strategy for ABCB1 inhibitors, by screening current FDA-approved drugs for ABCB1 inhibitory activity and establishing the usefulness of ABCB1 as a biomarker in PCa. We screened a library of FDA-approved drugs to find a compound that could revert drug-resistance in the DTX-resistant cell lines and found that ITZ, a triazole drug broadly used in treatment of a wide range of fungal infections, could revert DTX resistance in LNCaP^R^ and C4-2B^R^ (Figures 2B,C, respectively). This effect was observed in other DTX-resistant PCa cells, and independently of AR expression, illustrated by the PC-3^R^ and DU145^R^ cell lines (Figures 2D,E, respectively), and in DTX-resistant breast cancer cell lines, such as MDA-MB-231^R^ (Figure 3B). However, this effect was not observed for all DTX-resistant cell lines, as ITZ was unable to revert resistance in the BT-483, HCC202, HCC1569, and HCC1419 cell lines (Figure 3) at the concentrations of DTX we tested. Given the DTX IC_50_ values for these cell lines, and the magnitude of the effect that ITZ showed reverting resistance to DTX in the cell lines we tested (Figure 2), we would expect to see some reversal of DTX resistance, but it is possible that ITZ can partially revert DTX resistance in an ABCB-1 independent manner. These lines are resistant to DTX but showed no detectable expression of ABCB1 (Figure 3C, lower right subpanel), suggesting ITZ reversal of DTX resistance to be dependent of ABCB1 function. However, this is not an absolute requirement as we found that ITZ was able to confer sensitivity to DTX in the RCC-FG2 cell line (Figure 3), which does not express ABCB1. Moreover, we also found that ITZ could not revert DTX resistance in the ABCB1-overexpressing DTX-resistant breast cancer cell line, MCF-7^R^ (Figure 3A).

How to rationalize these findings in view of ABCB1-dependent mechanism for ITZ? These data do support the assertion that ITZ can only effectively circumvent ABCB1-associated DTX resistance, since the MCF-7^R^ cell line has a biphasic response pattern to DTX, with multiple resistance mechanisms mediating resistance to DTX, and ABCB1 inhibition with the prototypic ABCB1 inhibitor PSC-833 (valspodar) was also unable to revert DTX resistance at the concentrations of DTX that we used (Hansen et al., 2015; Hansen et al., 2016), paralleling our own results with ITZ. With respect to RCC-FG2, this clear cell renal cell carcinoma line showed an effect of ITZ, even though it does not express ABCB1, indicating that an additional effect of ITZ may be at play. Human clear cell renal cell carcinoma (RCC) is generally resistant to therapies, but the Hh signaling pathway is reactivated in RCC and was suggested to be an effective therapeutic target in RCC (Dormoy et al., 2009). ITZ is a known Hh inhibitor (Kim et al., 2010), which could explain its effect in RCC-FG2 cells. Unlike other azole antifungal drugs, ITZ does not block gonadal and adrenal steroidogenesis, suggesting it may potentially have value as a non-castrating treatment approach, but clinical studies of ITZ in biochemically recurrent prostate cancer, showed that ITZ had only a modest effect (Lee et al., 2019). These data is in agreement with our observation that ITZ on its own had little cytotoxicity with only a very modest effect observed at higher concentrations (Figure 2A). Since ITZ is a potent inhibitor of the hedgehog (Hh) signaling pathway (Kim et al., 2013; Inoue et al., 2016), and the Hh pathway effector transcriptional factor GLI1 regulates expression of ABCB1 (Chen et al., 2014), it was conceivable that ITZ could downregulate expression of ABCB1 through inhibition of Hh in the DTX-resistant PCa cells, but we found no effect of ITZ on steady state levels of ABCB1 protein expression in any of the cell lines tested (illustrated for C4-2B^R^ in Figure 4A), be it after 48 h of exposure to ITZ or to a combination of ITZ (2.5 µM) and DTX (100 nM) for up to 8 h (Figure 4A). Given that ABCB1 has a reported half-life of 5–18 h, and the timeframe of our analysis we cannot exclude that ITZ may eventually affect ABCB1 expression, but it is not the underlying mechanism behind the observed increase in intracellular concentration of DTX. *In vivo* imaging with fluorescently labelled docetaxel in C4-2B^R^ and LNCaP^R^ cells showed that ITZ can revert the increased efflux of docetaxel in these cells caused by overexpression of ABCB1 (Figure 4D through Figure 4G). Molecular docking of ITZ to ABCB1 indicated that itraconazole binds tightly to the inward-facing form of the transporter thereby preventing extrusion of DTX and causing intracellular retention of DTX (illustrated in Figure 5). Overall, our data indicates that ITZ can revert ABCB1-mediated DTX resistance in prostate cancer cells, through inhibition of ABCB1. There is some clinical evidence that treatment with ITZ, in combination with taxanes, can overcome chemoresistance in refractory disease. In ovarian cancer, adjunctive ITZ with a taxane-based chemotherapy significantly improved overall survival (HR = 0.27; *p* = 0.006) and progression-free survival (HR = 0.24; *p* = 0.002) (Tsubamoto et al., 2014).

One of the main factors that has negatively affected the clinical development of ABCB1 inhibitors is the development of unmanageable toxicities (Shukla et al., 2011). Early generation ABCB1 inhibitors failed clinical development due to severe side effects combined with a lack of potency and target selectivity, but even more specific third generation inhibitors that are generally well tolerated show dose-limiting toxicity. We used a repurposing strategy to identify a candidate drug with a favorable safety profile, and identified ITZ, a triazole broadly used for systemic treatment of a wide range of fungal infections. ITZ is well-tolerated, and most side effects are manageable and reversible. How to reconcile toxicity from ABCB1 inhibitors with the rather favorable safety profile of ITZ? One possible explanation comes from our observation that under the conditions we used, ITZ did not increase rho123 accumulation in DTX-resistant cells, thus suggesting that ITZ did not inhibit ABCB1-mediated efflux of rho123 (Figures 4A,B). Others have reported similar observations, with ITZ showing differential ABCB1 activity inhibition depending on substrates (Wang et al., 2002). ABC efflux pumps can be inhibited by compounds that either block drug binding site(s) or interfere with ATP hydrolysis of ABC proteins. Given our molecular docking results (Figure 5), it is likely that ITZ blocks drug-binding site(s), and given that ITZ did not inhibit ABCB1-mediated efflux of rho123, it probably blocks only specific sites. It does not interact with the R-site (rho123 binding site), but it does so with the site binding DTX. This substrate specific inhibition of ABCB1 may explain the lack of toxicity of ITZ.

Besides showing dose-limiting toxicity, ABCB1 inhibitors also failed to show clinical benefit. This may simply be because clinical studies of ABCB1 inhibitors did not routinely include molecular characterization of ABCB1 expression status in tumour tissues, which is a major issue since only a small fraction of cancers will express ABCB1 at functional levels capable of conferring drug resistance, and thus be expected to gain benefit from an ABCB1 inhibitor (Robey et al., 2018). We investigated the possible use of ABCB1 as a biomarker for DTX resistance in PCa, and as predictive biomarker for ITZ. In this study we used samples from chemo naïve PCa patients to assess MDR1 expression, to evaluate its possible use as a predictive biomarker in PCa treatment. However, all 315 prostate cancer tissue samples from patients who underwent radical prostatectomy we examined were negative for ABCB1 expression, suggesting increased levels of ABCB1 expression occur as a resistance mechanism only after exposure to DTX. A recent gene expression study, analyzing breast cancer tumors pre- and post-neoadjuvant anthracycline and taxane-based treatment, showed that ABCB1 expression is induced primarily upon treatment (Vera-Ramirez et al., 2013). We also previously demonstrated that DTX resistant sublines LNCaP^R^ and C4-2B^R^ only expressed ABCB1 when exposed to docetaxel, and during establishment of DTX resistance (Lima et al., 2021). Taken together, our data suggest that despite the importance of ABCB1 in multidrug resistance, its expression cannot be utilized as a biomarker to identify those prostate cancer patients that may be resistant to DTX prior to chemotherapy. And given the current clinical framework for prostate cancer, where raising prostate-specific antigen (PSA) is commonly used as a biochemical surrogate of clinical recurrence, and tissue biopsies are not routinely collected, repurposing of an already approved drug like ITZ, which can potentiate DTX chemotherapy, and overcome ABCB1-mediated drug resistance without adding severe toxicity may be a viable strategy.

In conclusion, available preclinical and clinical data indicate that ITZ can reverse taxane chemoresistance. Considering that PCa patients frequently present taxane-chemoresistant recurrences, and the limited availability of effective treatments for these patients, use of ITZ in an adjunctive setting warrants further study.

## Data Availability

The original contributions presented in the study are included in the article/supplementary material, further inquiries can be directed to the corresponding author.
